# Ethnic differences in perceptions of body satisfaction and body appearance among U.S. Schoolchildren: a cross-sectional study

**DOI:** 10.1186/1471-2458-12-425

**Published:** 2012-06-12

**Authors:** Rafael T Mikolajczyk, Ronald J Iannotti, Tilda Farhat, Vijaya Thomas

**Affiliations:** 1 Department of Clinical Epidemiology, Bremen Institute for Prevention Research and Social Medicine, Bremen, Germany; 2 Division of Epidemiology, Statistics and Prevention Research, Eunice Kennedy Shriver National Institute of Child Health and Human Development, Bethesda, USA; 3 Eunice Kennedy Shriver National Institute of Child Health and Human Development, 6100 Executive Blvd Room 7B13Q, MSC 7510, Bethesda, MD, 20892-7510, USA

## Abstract

**Background:**

Perceived body appearance and body satisfaction are potentially related to weight problems and poor health. The purpose of this study was to examine how gender, and ethnic differences in body satisfaction, perceived body appearance and weight status change by age in a representative sample of U.S. adolescents 11–17 years old.

**Methods:**

We used the US Health Behavior in School-Aged Children (HBSC) 2001 survey which assessed perceived body appearance, body satisfaction, self-reported body mass index (BMI) and socio-demographic indicators. The associations between age and perceived appearance, age and body satisfaction, and between z-transformed BMI and body satisfaction were analyzed using separate non-parametric regression models for both genders and the three ethnic groups.

**Results:**

Body satisfaction did not vary significantly by age except for an increase with age in the proportion of Non-Hispanic White girls who perceived themselves as too fat. Although boys did not report being too fat unless their BMI was above the age- and gender-specific median, one third of Non-Hispanic White girls felt too fat at or below the age- and gender-specific median. Compared to other ethnicities, African-American students’ perceived appearance was significantly more positive and they were less likely to perceive themselves overweight at higher BMI scores. However, during adolescence, the positive self-reported perceived appearance of African-American boys dropped substantially while it remained relatively stable in African-American girls.

**Conclusions:**

There were substantial differences in body satisfaction and perceived appearance across the three largest ethnic groups of school-age children in the U.S. Stability across age indicates that these perceptions are most likely established before the age of 10 and underline the importance of primary schools and parents in prevention. Special attention should be directed to the dramatic loss of positive perceived appearance among African-American boys.

## Background

Concerns with body weight and physical appearance are prevalent among adolescents [1,2] and are part of the larger phenomenon of body image perceptions, which relate to how individuals feel about their bodies [3]. Poor body image is often correlated with increasing body weight [4-6] and is linked to adverse outcomes such as psychological problems (including depression and low esteem) [4], perceived negative physical and mental health [7,8] and a higher likelihood of poor dieting and eating disorders, which may be sustained in adulthood [9]. Poor body image is also a potential mediator of the relationship between obesity and psychological distress [4]. While eating disorders generally tend to decline during the transition to early adulthood, poor body image remains a problem for a substantial segment of the adult population [10].

There is a large body of evidence addressing the differences in weight, weight perceptions, satisfaction with weight and their links with perceived appearance and attractiveness across ethnic groups in multiethnic populations like those in the USA (e.g. [11-19]) or the UK [20]. While most studies reported cross-sectional differences for specific age groups, some longitudinal studies were identified that addressed changes over time within predefined age ranges (e.g. [21-26]). However, the genesis of these differences and their development across the age spectrum remain insufficiently understood. Large variations in individuals’ weight and weight perceptions occur throughout the life course and are shaped by multiple factors. Newborns of different ethnic backgrounds can differ in their birthweight, based on genetic and phenotypical parental differences [27,28]. Parental and children’s own behaviors join to influence weight and weight perceptions in early childhood and kindergarten years. During the school years, interactions with peers and comparisons with peers may become the dominating reference system for children’s evaluations of their weight and their weight perceptions. Children’s media consumption also increases during this period and can contribute to perceptions of weight, appearance and attractiveness [29]. However, the age at which perceptions become stable or change is understudied.

In this study, our goal was to examine ethnic, sex and age differences in body satisfaction and perceptions of physical appearance versus body mass index (BMI) among a nationally representative sample of U.S. youth aged 11 to 17 years. The original study oversampled ethnic minorities, so we had a sufficient sample size to examine these relationships among Non-Hispanic Whites, African-American and Hispanic youth separately. We hypothesized that a) while body satisfaction and perceptions of physical appearance can be already established before the bottom end of the here studied age range, there will be substantial changes thereafter (conceptualized as differences by age), b) females will display stronger age effects, c) there will be particularly strong age effects among Non-Hispanic Whites.

## Methods

### Sample

The 2001 Health Behavior in School-Aged Children (HBSC) was a collaborative cross-sectional survey involving 36 countries in coordination with the World Health Organization [30]. The main goals of the survey were to assess the prevalence of health behaviors and to gain insight into the influence of the social context on young people’s well-being. The present paper analyzed data from the HBSC anonymous survey of a representative national sample of U.S. students in grades 6 through 10 during the 2001/2002 school year. Sampling was stratified by grade, with public, private and parochial schools selected to assure geographic representation and random selection of classrooms within grades. The sample was designed to provide estimates of prevalence within 3 percent at 95 percent confidence with sample design effects approximately 1.4 times greater than that of a simple random sample. An over-sampling of African-American and Hispanic students was included to provide better population estimates for these minorities. Five hundred forty-eight schools were selected to participate and 340 (62.5 percent) responded, yielding 18,593 eligible students. Parental consent and youth assent were required for participation in the survey. Of the eligible students (after eliminating 637 absent on the day of the survey, 600 not providing consent when required, 518 whose parents declined their child permission, and 1,620 students declining to participate), 15,245 (82 percent) completed questionnaires. Of this sample, 62 students who had missing data on a significant number of key items and 365 students who were outliers (< 1 percent ) for age in grade were dropped, leaving a sample of 14,818. Only those students in three ethnic groups (African-American, Non-Hispanic Whites and Hispanics) and who had completed questions about body satisfaction and perception of body appearance were included in the analysis resulting in the final sample size of 13,267. The Institutional Review Board at the *Eunice Kenney Shriver* National Institute of Child Health and Human Development approved the 2001 survey. Surveys were administered in classrooms by the class teacher. Questionnaires were completed anonymously and placed in a sealed envelope to ensure confidentiality.

### Variables

#### Perceived appearance

General body appearance was measured with a single question: “Do you think you are …?” with a five-point response scale: *very good looking*, *quite good looking*, *about average*, *not very good looking*, *not at all good looking*. A dichotomous variable was created with ‘very good looking’ versus the four remaining categories.

#### Body satisfaction

Body satisfaction was measured with the question: “Do you think your body is…?” with a five-point response scale: *much too thin, a bit too thin, about the right size, a bit too fat, much too fat*. For the analysis, the variable was dichotomized into too fat (a bit too fat and much too fat) versus the three remaining categories.

#### Body mass index (BMI)

BMI was obtained from self-reported weight and height. BMI was converted into age- (in months) and gender-standardized z-transformed values using values derived from the NHANES study [31]. Thus the unit of the new variable (BMIZ) is one standard deviation of the BMI distribution. Values within one standard deviation correspond to 68% of the original distribution, while values within 2 units correspond to 95%, and those within 3 units to 99.7% of the original distribution. The advantage of this conversion is that values of the BMIZ variable can be compared across age and gender.

#### Demographic characteristics

Students provided demographic information about race/ethnicity (Hispanic/Latino, Non-Hispanic Black, and Non-Hispanic White) and date of birth. Age was calculated in years from the date of birth and date of the completion of the questionnaire and rounded to one decimal place. The highest level of education of mother or father reported by the child was used as an index of parent education. The Family Affluence Scale (FAS) assessed the number of family cars, vacations in the past year, home computers, and whether the respondent had his or her own bedroom. Studies indicate the scale has good content validity and external reliability [32]. Children also indicated whether they lived in an “urban area (city)”, “suburban area (near a large city)” or “rural area (not near a large city).”

### Statistical analysis

Sample descriptive statistics (demographic characteristics) were compared across ethnic groups using chi-square tests. The association between age and perceived appearance was analyzed using separate non-parametric regression models for both genders and the three ethnic groups. Age was treated as a continuous variable and locally-weighted regression [33,34] was used to assess the form of the association. Locally-weighted regression uses data points in the immediate neighborhood to derive the estimate of the given data point without making assumptions about the general form of the association. We used the *gam* library in R to apply this model. Non-parametric regression models were also used to assess the association between age and body satisfaction, and between z-transformed BMI and body satisfaction. Significance level was set at p < 0.05 for all analyses.

## Results

### Description of the sample

Gender and age distributions were similar across the three ethnic groups, but the proportion of children from high-affluent families was much higher in Non-Hispanic Whites (Table 1). Parental education was highest in Non-Hispanic Whites, followed by African-American and (substantially lower) Hispanics. More than half of Non-Hispanic White adolescents lived in suburban and rural areas, while the majority of Hispanic and African-American students lived in urban areas. As defined by the 90^th^ percentile of the national age- and gender-specific weight standard, Non-Hispanic White boys and girls had substantially less obesity than boys and girls in the other two racial/ethnic groups (Table 2). Body appearance was rated highest by African-American boys and girls (Table 2). Non-Hispanic Whites and Hispanics rated their appearance similarly, but substantially lower than African-Americans. Again, the results were similar for both genders. Conversely, Non-Hispanic Whites and Hispanics indicated dissatisfaction with their body weight (being “too fat”) more often than African-Americans (Table 2).

**Table 1:** **Description of the sample by ethnic group (%)**^**a**^

**Variables**	**Hispanic**	**African-American**	**Non-Hispanic White**
	**N = 2869**	**N = 3017**	**N = 7381**
Age			
10.5-11.49	5.8	5.5	3.5
11.5-12.49	20.5	19.0	21.0
12.5-13.49	18.2	20.2	20.2
13.5-14.49	18.6	18.0	19.5
14.5-15.49	18.7	18.5	18.1
15.5-16.49	15.7	15.5	15.9
16.5-17.49	2.6	3.3	1.8
Gender			
Boys	47.4	44.7	48.3
Girls	52.6	55.3	51.7
Family Affluence			
Low	41.4	42.6	22.1
Moderate	47.1	43.9	53.9
High	11.5	13.5	24.0
Parental Education			
< High School	25.8	8.9	6.8
High School	22.4	26.2	20.6
> High School	21.1	22.1	21.6
College	30.7	42.9	51.1
Urbanicity			
Urban	58.8	61.8	28.5
Suburban	22.9	19.1	33.9
Rural	18.3	19.2	37.7

^a^ All comparisons across ethnic groups were significant (p < 0.05).

**Table 2:** **Body perception and body mass index by gender and ethnic background (%)**^**a**^

	**Boys**			**Girls**		
**Variables**	**Hispanic**	**African-American**	**Non-Hispanic White**	**Hispanic**	**African-American**	**Non-Hispanic White**
	**N = 1315**	**N = 1290**	**N = 3484**	**N = 1479**	**N = 1626**	**N = 3771**
BMI						
Missing	18.2	21.0	8.2	20.0	17.4	9.1
Reported	81.8	79.0	91.8	80.0	82.6	90.9
BMIZ						
<85th pct	60.7	68.1	72.0	72.0	67.6	82.3
≥85th pct	39.3	31.9	28.0	28.0	32.4	17.7
BMIZ						
<90th pct	70.1	75.0	79.2	80.1	75.7	87.7
≥90th pct	29.9	25.0	20.8	19.9	24.3	12.3
BMIZ						
<10th pct	6.6	6.5	7.9	6.6	5.2	9.8
≥10th pct	93.4	93.5	92.1	93.4	94.8	90.2
Appearance						
Very good	22.5	44.2	19.1	16.9	48.0	12.8
Quite good	24.3	25.3	24.2	26.3	25.0	25.4
About average	43.9	27.7	45.8	42.3	23.3	49.9
Not very good	6.4	1.6	7.2	10.3	2.4	8.3
Not at all good	2.9	1.2	3.6	4.3	1.3	3.5
Body Satisfaction						
Much too thin	2.4	3.6	2.8	2.7	3.2	1.4
A bit too thin	11.2	14.2	13.6	8.4	10.8	7.3
About the right size	54.8	62.7	57.2	49.5	56.9	51.7
A bit too fat	28.5	16.9	22.7	33.7	24.5	33.8
Much too fat	3.0	2.7	3.6	5.8	4.7	5.7

^a^ All comparisons across ethnic groups were significant (p < 0.05).

### Differences in perceived appearance and body satisfaction across age

**Figure 1:**
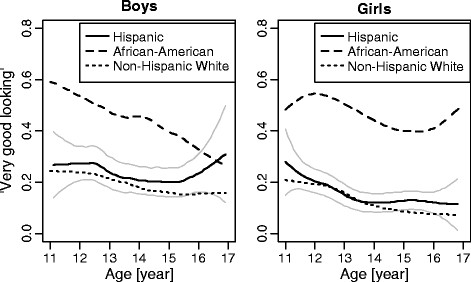
**Proportion of children and adolescents by gender, age and ethnic group who considered themselves to be very good looking (locally weighted regression with age treated as continuous variable, point-wise 95% confidence intervals are presented for Hispanics). **Note: With the exception of the trend for African-American girls, there was a significant negative linear trend for all gender by ethnicity subgroups (p < 0.05).

The proportion of children who consider themselves very good looking decreased with age in both genders and all three ethnic groups with the exception of African-American girls (Figure 1). The patterns of change across age did not differ between Hispanic and Non-Hispanic White youths, but was significantly different in African-American youths. There was also an important difference between African-American girls and boys: while the proportion of African-American girls considering themselves very good looking fluctuated consistently at a higher level than the other groups, African-American boys experienced a substantial decrease from nearly 60% who considered themselves good-looking at the age of 11 years to around 30% at the age of 17. By the age of 17, after this dramatic fall in the percent of African-American boys who considered themselves good looking, no differences were observed between African-American boys and those from the two other male ethnic groups. Non-Hispanic White and Hispanic older adolescent girls displayed an overly pessimistic self-perception.

**Figure 2:**
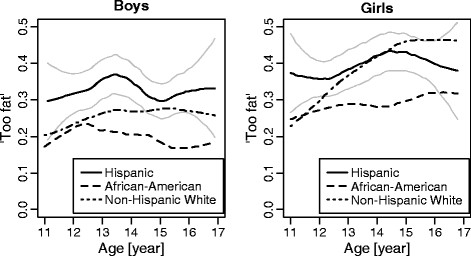
**Proportion of children and adolescents by gender, age and ethnic group who considered themselves to be “much too fat” or “a bit too fat” (locally weighted regression with age treated as continuous variable, point-wise 95% confidence intervals are presented for Hispanics). **Note: Linear trend in logistic regression was significant only for Non-Hispanic White girls (p < 0.05).

In contrast to the perception of appearance, body satisfaction did not differ over the age range studied except for Non-Hispanic White girls (Figure 2). The proportion of Non-Hispanic White girls considering themselves too fat nearly doubled between 11 and 15 years of age, but was stable from ages 15 to 17.

### Reported BMI and body satisfaction

**Figure 3:**
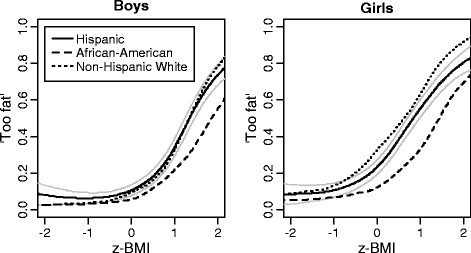
**Fraction of children or adolescents considering themselves “too fat” by reported BMI (z-transformed, see methods), gender and ethnic group (locally weighted regression with z-transformed BMI treated as continuous variable, point-wise 95% confidence intervals are presented for Hispanics as gray solid lines). **Note: z-BMI is z-transformed BMI; y-axis indicates the proportion of those responding “too fat” among all children of the given BMI.

The proportion of boys who perceive themselves as too fat started to increase gradually when the reported BMI corresponded to the mean value of the sample (0 on the z-transformed scale) (Figure 3 ). At one standard deviation above the mean (which corresponds to a BMI greater than the 84^th^ percentile of the population) approximately one third of the boys considered themselves too fat. This increased to 70% or more at two standard deviations (a BMI greater than approximately 98% of the population). Non-Hispanic White and Hispanic boys did not differ in the relationships between weight perceptions and BMI, but African-American boys were less likely to consider themselves too fat at the same BMI percentiles. This tendency was even stronger among African-American girls; however among girls there was also a difference between Non-Hispanic Whites and Hispanics, with the former starting to label themselves as too fat at lower BMI percentiles. The curves also shifted towards lower BMI values for Hispanic and Non-Hispanic White girls in comparison to boys, indicating more weight concerns among girls than boys at the same age- and gender-adjusted BMI percentiles.

The results were qualitatively similar regarding the comparison between ethnic groups for the ‘about the right size’ weight perception category (data not shown). The proportion of children/adolescents who still considered their weight about right when their reported BMI was above the 90^th^ percentile was highest among African-American boys and girls but also substantial among other groups. In comparison, at very low BMI values, large proportions of children in all groups considered themselves about right.

## Discussion

We analyzed a representative sample of U.S. schoolchildren with respect to body satisfaction and perceived appearance. All three assessed ethnic groups displayed distinct characteristics. For Non-Hispanic Whites and Hispanics, perceptions of body appearance were similar for both genders and across the age spectrum, but African-American youths differed substantially from both other groups. African-American youths had much better perceived body appearance at the youngest age. Perceived appearance remained high in African-American girls over the age range, but dropped dramatically in African-American boys. There was also a non-negligible drop in positive perceived appearance among Non-Hispanic White and Hispanic girls towards the end of the analyzed age spectrum (older adolescence). Only Non-Hispanic White girls exhibited an increase in the proportion considering themselves too fat; the proportion doubled between 11 and 15 years of age. (In this age group BMI scores also increased among Non-Hispanic White girls.) Conversely, perceptions of weight remained stable for all other groups. Non-Hispanic White and Hispanic boys had similar perceptions of body weight in relation to their BMI, compared to African-American boys, who were less likely to perceive themselves as too fat at higher BMI percentiles. Girls in all three ethnic groups considered themselves as being too fat at substantially lower age- and gender-specific percentiles of BMI than boys. However, there was a substantial difference across all three ethnic groups, with Non-Hispanic White girls substantially more likely to perceive themselves too fat at a given BMI percentile, followed by Hispanics and African-American.

The perceived positive appearance in African-American boys was exceptionally high at young ages (60% considered themselves being very good looking), but the dramatic drop between 11 to 17 years of age is nevertheless troubling. A longitudinal study in Finland found that perceptions regarding appearance are increasingly fixed with age and more stable in boys than girls [35], thus underscoring the importance of the unusual process occurring in African-American boys. The drop in perceived positive appearance in African-American boys may be associated with increasing environmental awareness, which develops during adolescence and may partially account for the decrease in self-perception [36]. McCabe and Ricciardelli [37] report that, as boys get older, “they become more aware of the sociocultural ideal for males, and they recognize that there is pressure to strive not only for a larger body, but also for a body that demonstrates muscular strength and tone” (p.676). This could be especially true for African-American boys since research shows that they are at greater risk for weight-related concerns and behaviors, as compared to Non-Hispanic Whites [38]. Paradoxically, the excessive weight-related concerns among this group could stem from the disconnect between the larger ideal body sizes that they strive for [39] and their perception that they are not big enough.

African-American girls showed high self-perception at the age of 11, and sustained it across all of the ages studied. In contrast, there was a substantial loss of positive self-perception among Non-Hispanic White and Hispanic girls towards the end of the studied age spectrum. There is evidence that the negative relationship between BMI and perceived physical appearance is greater in Non-Hispanic White girls ages 9 to 14, compared to African American girls [6]. There is also research indicating that body sizes presented in the media differ by ethnicity and thus media can influence perceived appearance across ethnic groups in different intensity [40]. Therefore, the observed effects among girls could be either linked to the increase of BMI or to the increased exposure to the media.

In all but Non-Hispanic White girls, proportions of children perceiving themselves as too fat did not differ by age in the studied range (11 to 17 years). In earlier studies, pre-adolescents have been excluded from studies on body image because it was thought that these issues mostly affect adolescents [41,42]. However, our findings agree with more recent studies that challenge this perspective and suggest that body image concerns are increasingly affecting pre-adolescents [43]. The percentage of Non-Hispanic White girls who considered themselves much too fat or a bit too fat increased between 11 and 15 years of age, and then leveled off between 15 and 17 years. This differential effect may be the result of different socio-cultural factors such as the media, peers, and parents [44]. These results are consistent with findings that African-American and Hispanic females respond differently to Non-Hispanic White media images than their Non-Hispanic White counterparts, and possibly reject the implicit beauty and social pressure to match this “thin” ideal, which Non-Hispanic White girls internalize [45-47]. African-American and Hispanic girls may also perceive that their peers and parents have less weight concerns and larger ideal body sizes compared to their Non-Hispanic White counterparts [39,48,49]. Although further research is needed to explain the stabilization of body perception from 15 to 17 years of age for Non-Hispanic White girls, one possible explanation may be that social comparisons and cultural expectations have already been internalized by age 15 [36]. Our analysis also demonstrated that the proportion of boys with a perception of being much too fat or a bit too fat did not change with age, which corroborates previous studies that socio-cultural factors perpetuate the muscular body ideal for males [50].

Early research on body satisfaction problems focused mainly on girls [51]; recently more attention has been paid to body image among boys [37,38,51]. Our findings indicate that while concerns regarding overweight are more frequent among girls, a substantial proportion of boys also considered their weight as too high. In addition, our findings indicate a higher proportion of boys considered themselves to be too thin. This may be another reflection of the muscular ideal which is more evident in boys [50]. Similar to women’s internalization of the culturally ideal thin body, men may be susceptible to internalizing a muscular ideal from the media [52].

Although African-American girls had the highest age- and gender-specific BMI compared to other ethnic groups and African-American boys had substantially higher BMI than Non-Hispanic White boys (and only slightly lower than Hispanic boys), African-American boys and girls were more tolerant than Non-Hispanic Whites and Hispanics regarding the BMI z-score at which they considered themselves too fat. This finding is consistent with a large body of literature indicating different ideals of body attractiveness in African-Americans [19,38,39,53,54] and a lower awareness of overweight among African-Americans [5,33,55]. The finding is double-edged: On the one hand, there is substantial evidence that obesity increases multiple health risks; greater weight concerns can support weight loss behavior or the maintenance of a low body weight, thereby subsequently reducing health risks associated with obesity. On the other hand, weight concerns can affect physical and psychological well-being or even misdirect weight-loss strategies [4,5,56]. These findings call for balanced strategies to promote healthy weight perception, with messages framed differently for each ethnic group. While addressing media pressure can be of utmost importance for Non-Hispanic White girls [40], nutritional and physical activity interventions can be more important for other groups [15,57]. For Hispanic adolescents the issue of acculturation was also raised, which additionally complicates the relationship between body perception and appearance [58,59] Addressing the links between poor body image and eating disorders might require psychosocial interventions as suggested by the research [11].

### Limitations

This study is based on cross-sectional data, while its main interest is in changes across age. However, it is unlikely that there are cohort effects across such a narrow age range. Despite previous research on the stability of body image perceptions, we do not know how stable these perceptions are within individuals over shorter time periods. While one might think that body weight perceptions do not change from day to day, perceived appearance could be affected by concurrent social interactions. The measures related to perception were subjective per se, but BMI could have been objectively measured. Instead we had to rely on BMI calculated from self reported weight and height. Previous research indicated the self-reported BMI correlates well with measured BMI, in a way which can be sufficient for studies of associations [60-63]. Nevertheless, self-reported BMI may be systematically biased not only for the population, but even more within specific subgroups, but is an adequate measure if no other possibilities exist [64].

With the exception of the three groups studied, our sample of other ethnic groups was too small for reliable estimates of effects. Furthermore, information about BMI was missing for a substantial fraction of students. While missing information might simply reflect that the child does not know his/her current weight, it could also indicate that he/she is not as occupied with the issue of body weight. Therefore, excluding children with missing self-reported BMI might have resulted in a sample of children more concerned with their weight.

When calculating standardized BMI values we used a common standard for all three ethnic groups and did not ask what respondents considered the reference for their weight. This ignores the fact that the own ethnic group can be the reference for body image and thus individual perceptions of overweight can be based on ethnic-specific standards.

While dichotomizing variables we had to accept some loss of information, but at the same time applying ANOVA would violate assumptions of normal data distribution. We also contrasted the highest values with the remaining values, in this way we focused on the upper end of the spectrum of responses and did not report the findings for those with negative body appearance.

## Conclusions

There are substantial differences in perceived appearance and body satisfaction across the three largest ethnic groups of school-age children in the U.S.: While Non-Hispanic Whites and Hispanics have similar perceptions about body image, both groups differ from African-Americans. For many adolescents perceived appearance and body satisfaction are most likely established before the age of 10, with little change during adolescence. Since both perceptions are potentially related to weight problems and poor health, their early stability underlines the importance of primary schools and parents in prevention. Special attention should be targeted to the dramatic loss of positive perceived appearance among African-American boys in the analyzed age spectrum. It is also important to note the rather negative self-perception among Non-Hispanic White and Hispanic girls, especially towards the upper end of the age spectrum.

## Competing interests

The authors declare that they have no competing interests.

## Authors’ contributions

Conception and design of the study: RTM. Acquisition of the data: RJI; Analysis of the data, RTM; Interpretation of the data RTM, RJI, and TF; Writing of the manuscript, RTM, RJI, TF and VT. All authors gave final approval of the version to be published. All authors read and approved the final manuscript.

## Pre-publication history

The pre-publication history for this paper can be accessed here:

http://www.biomedcentral.com/1471-2458/12/425/prepub

## References

[B1] Neumark-Sztainer D Hannan PJ Weight-related behaviors among adolescent girls and boys: results from a national survey Arch Pediatr Adolesc Med 2000 154 6 569 577 10850503 10.1001/archpedi.154.6.569

[B2] Phelps L Johnston LS Jimenez DP Wilczenski FL Andrea RK Healy RW Figure Preference, Body Dissatisfaction, and Body Distortion in Adolescence J Adolesc Res 1993 8 297 310 10.1177/074355489383005

[B3] Ata RN Ludden AB Lally MM The effects of gender and family, friend, and media influences on eating behaviors and body image during adolescence J Youth Adolesc 2007 36 1024 1037 10.1007/s10964-006-9159-x

[B4] Friedman KE Reichmann SK Costanzo PR Musante GJ Body image partially mediates the relationship between obesity and psychological distress Obes Res 2002 10 1 33 41 10.1038/oby.2002.5 11786599

[B5] Young-Hyman D Tanofsky-Kraff M Yanovski SZ Keil M Cohen ML Peyrot M Yanovski JA Psychological status and weight-related distress in overweight or at-risk-for-overweight children Obesity (Silver Spring) 2006 14 12 2249 2258 10.1038/oby.2006.264 17189553 PMC1862955

[B6] Brown KM McMahon RP Biro FM Crawford P Schreiber GB Similo SL Waclawiw M Striegel-Moore R Changes in self-esteem in black and white girls between the ages of 9 and 14 years. The NHLBI Growth and Health Study J Adolesc Health 1998 23 1 7 19 10.1016/S1054-139X(97)00238-3 9648018

[B7] Meland E Haugland S Breidablik HJ Body image and perceived health in adolescence Health Educ Res 2007 22 3 342 350 16957015 10.1093/her/cyl085

[B8] Muennig P Health selection vs. causation in the income gradient: what can we learn from graphical trends? J Health Care Poor Underserved 2008 19 2 574 579 10.1353/hpu.0.0018 18469427

[B9] Moore DC Body image and eating behavior in adolescents J Am Coll Nutr 1993 12 5 505 510 8263264 10.1080/07315724.1993.10718343

[B10] Heatherton TF Mahamedi F Striepe M Field AE Keel P A 10-year longitudinal study of body weight, dieting, and eating disorder symptoms J Abnorm Psychol 1997 106 1 117 125 9103723

[B11] Ceballos N Czyzewska M Body image in Hispanic/Latino vs. European American adolescents: implications for treatment and prevention of obesity in underserved populations J Health Care Poor Underserved 2010 21 3 823 10.1353/hpu.0.0333 20693729

[B12] Grabe S Hyde JS Ethnicity and body dissatisfaction among women in the United States: a meta-analysis Psychol Bull 2006 132 4 622 16822170 10.1037/0033-2909.132.4.622

[B13] Gillen MM Lefkowitz ES Emerging adults’ perceptions of messages about physical appearance Body Image 2009 6 3 178 10.1016/j.bodyim.2009.02.002 19410527

[B14] Lynch WC Heil DP Wagner E Havens MD Ethnic differences in BMI, weight concerns, and eating behaviors: comparison of Native American, White, and Hispanic adolescents Body Image 2007 4 2 179 10.1016/j.bodyim.2007.01.001 18089263 PMC2031858

[B15] Neumark-Sztainer D Croll J Story M Hannan PJ French SA Perry C Ethnic/racial differences in weight-related concerns and behaviors among adolescent girls and boys: findings from Project EAT J Psychosom Res 2002 53 5 963 10.1016/S0022-3999(02)00486-5 12445586

[B16] Perry AC Rosenblatt EB Wang X Physical, behavioral, and body image characteristics in a tri-racial group of adolescent girls Obes Res 2004 12 10 1670 10.1038/oby.2004.207 15536231

[B17] Talpade M Hispanic versus African American girls: body image, nutrition, and puberty Adolescence 2008 43 169 119 18447084

[B18] Yanover T Thompson JK Perceptions of health and attractiveness: the effects of body fat, muscularity, gender, and ethnicity J Heal Psychol 2010 15 7 1039 10.1177/1359105309360426 20511287

[B19] Roberts A Cash TF Feingold A Johnson BT Are black-white differences in females’ body dissatisfaction decreasing? A meta-analytic review J Consult Clin Psychol 2006 74 6 1121 1131 17154741 10.1037/0022-006X.74.6.1121

[B20] Higgins V Dale A Ethnicity and childhood overweight/obesity in England Pediatric Obes 2012 7 3 e22 e26 Epub 2012 Apr 11. 10.1111/j.2047-6310.2012.00051.x 22495944

[B21] Gillen MM Lefkowitz ES Gender and racial/ethnic differences in body image development among college students Body Image 2012 9 1 126 130 10.1016/j.bodyim.2011.09.004 21983339 PMC3246027

[B22] Datar A Shier V Sturm R Changes in body mass during elementary and middle school in a national cohort of kindergarteners Pediatrics 2011 128 6 e1411 1417 10.1542/peds.2011-0114 22106078 PMC3387897

[B23] Vogt Yuan AS Body perceptions, weight control behavior, and changes in adolescents’ psychological well-being over time: a longitudinal examination of gender J Youth Adolesc 2010 39 8 927 10.1007/s10964-009-9428-6 20596819

[B24] Neumark-Sztainer D Paxton SJ Hannan PJ Haines J Story M Does body satisfaction matter? Five-year longitudinal associations between body satisfaction and health behaviors in adolescent females and males J Adolesc Health 2006 39 2 244 251 10.1016/j.jadohealth.2005.12.001 16857537

[B25] Paxton SJ Eisenberg ME Neumark-Sztainer D Prospective predictors of body dissatisfaction in adolescent girls and boys: a five-year longitudinal study Dev Psychol 2006 42 5 888 899 16953694 10.1037/0012-1649.42.5.888

[B26] Vogt Yuan AS Body perceptions, weight control behavior, and changes in adolescents’ psychological well-being over time: a longitudinal examination of gender J Youth Adolesc 2010 39 8 927 939 Epub 2009 Jun 25. 10.1007/s10964-009-9428-6 20596819

[B27] Gardosi J Ethnic differences in fetal growth Ultrasound Obstet Gynecol 1995 6 2 73 74 10.1046/j.1469-0705.1995.06020073.x 8535920

[B28] Mikolajczyk RT Zhang J Betran AP Souza JP Mori R Gulmezoglu AM Merialdi M A global reference for fetal-weight and birthweight percentiles Lancet 2011 377 9780 1855 1861 10.1016/S0140-6736(11)60364-4 21621717

[B29] Grabe S Ward LM Hyde JS The role of the media in body image concerns among women: a meta-analysis of experimental and correlational studies Psychol Bull 2008 134 3 460 18444705 10.1037/0033-2909.134.3.460

[B30] Currie C Roberts C Morgan A Young people’s health in context - Health Behaviour in School-aged Children (HBSC) study: International report from the 2001/02 survey 2004 Kopenhagen: WHO-Europe eds.

[B31] CDC Growth Charts: United States [http://www.cdc.gov/nchs/about/major/nhanes/growthcharts/datafiles.htm].

[B32] Currie C Molcho M Boyce W Holstein B Torsheim T Richter M Researching health inequalities in adolescents: the development of the Health Behaviour in School-Aged Children (HBSC) family affluence scale Soc Sci Med 2008 66 6 1429 1436 10.1016/j.socscimed.2007.11.024 18179852

[B33] Cleveland WS Grosse E Shyu WM Chambers JM, Hastie TJ Local regression models Statistical models in S 1992 acific Grove, CA: Wadsworth/Brooks-Cole 309 376

[B34] Hastie TJ Chambers JM, Hastie TJ Generalized additive models Statistical Models in S 1991 Pacific Grove, CA: Wadsworth & Brooks/Cole Chapter 7

[B35] Lintunen T Leskinen E Oinonen M Salinto M Rahkila P Change, Reliability, and Stability in Self-Perceptions in Early Adolescence - A 4-Year Follow-Up-Study Int J Behav Dev 1995 18 351 364 10.1016/0163-6383(95)90023-3

[B36] Morrison TG Kalin R Morrison MA Body-image evaluation and body-image investment among adolescents: a test of sociocultural and social comparison theories Adolescence 2004 39 155 571 592 15673231

[B37] McCabe MP Ricciardelli LA Body image dissatisfaction among males across the lifespan: a review of past literature J Psychosom Res 2004 56 6 675 685 10.1016/S0022-3999(03)00129-6 15193964

[B38] Neumark-Sztainer D Croll J Story M Hannan PJ French SA Perry C Ethnic/racial differences in weight-related concerns and behaviors among adolescent girls and boys: findings from Project EAT J Psychosom Res 2002 53 5 963 974 10.1016/S0022-3999(02)00486-5 12445586

[B39] Adams K Sargent RG Thompson SH Richter D Corwin SJ Rogan TJ A study of body weight concerns and weight control practices of 4th and 7th grade adolescents Ethn Health 2000 5 1 79 94 10.1080/13557850050007374 10858942

[B40] Shoneye C Johnson F Croker H Steptoe A Wardle J Body sizes in print media: Are there ethnic differences? A brief report Eat Weight Disord 2011 16 3 e212 215 22290039 10.1007/BF03325135

[B41] Blyth DA Simmons RG Zakin DF Satisfaction with Body-Image for Early Adolescent Females - the Impact of Pubertal Timing Within Different School Environments J Youth Adolesc 1985 14 207 225 10.1007/BF02090319 24301177

[B42] Sands R Tricker J Sherman C Armatas C Maschette W Disordered eating patterns, body image, self-esteem, and physical activity in preadolescent school children Int J Eat Disord 1997 21 2 159 166 10.1002/(SICI)1098-108X(199703)21:2<159::AID-EAT6>3.0.CO;2-K 9062839

[B43] Robinson TN Chang JY Haydel KF Killen JD Overweight concerns and body dissatisfaction among third-grade children: the impacts of ethnicity and socioeconomic status J Pediatr 2001 138 2 181 187 10.1067/mpd.2001.110526 11174614

[B44] Field AE Cheung L Wolf AM Herzog DB Gortmaker SL Colditz GA Exposure to the mass media and weight concerns among girls Pediatrics 1999 103 3 E36 10.1542/peds.103.3.e36 10049992

[B45] Rubin LR Fitts ML Becker AE “Whatever feels good in my soul”: body ethics and aesthetics among African American and Latina women Cult Med Psychiatry 2003 27 1 49 75 12825784 10.1023/a:1023679821086

[B46] Milkie MA Social comparisons, reflected appraisals, and mass media: The impact of pervasive beauty images on black and white girls’ self-concepts Soc Psychol Q 1999 62 190 210 10.2307/2695857

[B47] Knauss C Paxton SJ Alsaker FD Relationships amongst body dissatisfaction, internalisation of the media body ideal and perceived pressure from media in adolescent girls and boys Body Image 2007 4 4 353 360 10.1016/j.bodyim.2007.06.007 18089281

[B48] Winkleby MA Gardner CD Taylor CB The influence of gender and socioeconomic factors on Hispanic/white differences in body mass index Prev Med 1996 25 2 203 211 10.1006/pmed.1996.0047 8860286

[B49] Thompson S Sargent R Rogan T Corvin SJ Sociocultural influences on weight concerns among early adolescents J Gend Cult Heal 1997 2 211 231

[B50] Labre MP Adolescent boys and the muscular male body ideal J Adolesc Health 2002 30 4 233 242 10.1016/S1054-139X(01)00413-X 11927235

[B51] Cohane GH Pope HG Jr Body image in boys: a review of the literature Int J Eat Disord 2001 29 4 373 379 10.1002/eat.1033 11285574

[B52] Lorenzen LA Exposure to Muscular Male Models Decreases Men’s Body Satisfaction Sex Roles 2004 51 11/12 743 748

[B53] Parnell K Sargent R Thompson SH Duhe SF Valois RF Kemper RC Black and white adolescent females’ perceptions of ideal body size J Sch Health 1996 66 3 112 118 8857160

[B54] Wildes JE Emery RE Simons AD The roles of ethnicity and culture in the development of eating disturbance and body dissatisfaction: a meta-analytic review Clin Psychol Rev 2001 21 4 521 551 10.1016/S0272-7358(99)00071-9 11413866

[B55] Kimm SY Barton BA Berhane K Ross JW Payne GH Schreiber GB Self-esteem and adiposity in black and white girls: the NHLBI Growth and Health Study Ann Epidemiol 1997 7 8 550 560 10.1016/S1047-2797(97)00124-5 9408551

[B56] Davison TE McCabe MP Adolescent body image and psychosocial functioning J Soc Psychol 2006 146 1 15 30 10.3200/SOCP.146.1.15-30 16480119

[B57] Sanchez-Johnsen LAP Fitzgibbon ML Martinovich Z Stolley MR Dyer AR Van Horn L Ethnic Differences in correlates of obesity between Latin-American and black Women Obes Res 2004 12 4 652 10.1038/oby.2004.75 15090633

[B58] Gonzales-Backen MA Umana-Taylor AJ Examining the role of physical appearance in Latino adolescents’ ethnic identity J Adolesc 2011 34 1 151 10.1016/j.adolescence.2010.01.002 20167356

[B59] Olvera N Smith DW Lee C Liu J Lee J Kim J-H Kellam SF Comparing high and low acculturated mothers and physical activity in Hispanic children J Phys Act Heal 2011 8 Suppl 2 S206 213 21918234

[B60] Elgar FJ Roberts C Tudor-Smith C Moore L Validity of self-reported height and weight and predictors of bias in adolescents J Adolesc Health 2005 37 5 371 375 10.1016/j.jadohealth.2004.07.014 16227121

[B61] Goodman E Hinden BR Khandelwal S Accuracy of teen and parental reports of obesity and body mass index Pediatrics 2000 106 1 Pt 1 52 58 10878149 10.1542/peds.106.1.52

[B62] Sherry B Jefferds ME Grummer-Strawn LM Accuracy of adolescent self-report of height and weight in assessing overweight status: a literature review Arch Pediatr Adolesc Med 2007 161 12 1154 1161 10.1001/archpedi.161.12.1154 18056560

[B63] Brener ND McManus T Galuska DA Lowry R Wechsler H Reliability and validity of self-reported height and weight among high school students J Adolesc Health 2003 32 4 281 287 10.1016/S1054-139X(02)00708-5 12667732

[B64] Mikolajczyk RT Maxwell AE El Ansari W Stock C Petkeviciene J Guillen-Grima F Relationship between perceived body weight and body mass index based on self- reported height and weight among university students: a cross-sectional study in seven European countries BMC Publ Health 2010 10 40 10.1186/1471-2458-10-40 PMC2824734 20105333

